# Deficiency of arcuate nucleus kisspeptin results in postpubertal central hypogonadism

**DOI:** 10.1152/ajpendo.00088.2021

**Published:** 2021-06-28

**Authors:** Nimisha Nandankar, Ariel L. Negrón, Andrew Wolfe, Jon E. Levine, Sally Radovick

**Affiliations:** ^1^Department of Pediatrics, Child Health Institute of New Jersey, Rutgers-Robert Wood Johnson Medical School, Rutgers, the State University of New Jersey, New Brunswick, New Jersey; ^2^Division of Physiological and Pathological Sciences, National Institutes of Health, Bethesda, Maryland; ^3^Wisconsin National Primate Research Center, University of Wisconsin, Madison, Wisconsin

**Keywords:** arcuate nucleus, hypogonadism, kisspeptin, luteinizing hormone, reproduction

## Abstract

Kisspeptin (encoded by *Kiss1*), a neuropeptide critically involved in neuroendocrine regulation of reproduction, is primarily synthesized in two hypothalamic nuclei: the anteroventral periventricular nucleus (AVPV) and arcuate nucleus (ARC). AVPV kisspeptin is thought to regulate the estrogen-induced positive feedback control of gonadotropin-releasing hormone (GnRH) and luteinizing hormone (LH), and the preovulatory LH surge in females. In contrast, ARC kisspeptin neurons, which largely coexpress neurokinin B and dynorphin A (collectively named KNDy neurons), are thought to mediate estrogen-induced negative feedback control of GnRH/LH and be the major regulators of pulsatile GnRH/LH release. However, definitive data to delineate the specific roles of AVPV versus ARC kisspeptin neurons in the control of GnRH/LH release is lacking. Therefore, we generated a novel mouse model targeting deletion of *Kiss1* to the ARC nucleus (Pdyn-Cre/Kiss1^fl/fl^ KO) to determine the functional differences between ARC and AVPV kisspeptin neurons on the reproductive axis. The efficacy of the knockout was confirmed at both the mRNA and protein levels. Adult female Pdyn-Cre/Kiss1^fl/fl^ KO mice exhibited persistent diestrus and significantly fewer LH pulses when compared with controls, resulting in arrested folliculogenesis, hypogonadism, and infertility. Pdyn-Cre/Kiss1^fl/fl^ KO males also exhibited disrupted LH pulsatility, hypogonadism, and variable, defective spermatogenesis, and subfertility. The timing of pubertal onset in males and females was equivalent to controls. These findings add to the current body of evidence for the critical role of kisspeptin in ARC KNDy neurons in GnRH/LH pulsatility in both sexes, while directly establishing ARC kisspeptin’s role in regulating estrous cyclicity in female mice, and gametogenesis in both sexes, and culminating in disrupted fertility. The Pdyn-Cre/Kiss1^fl/fl^ KO mice present a novel mammalian model of postpubertal central hypogonadism.

**NEW & NOTEWORTHY** We demonstrate through a novel, conditional knockout mouse model of arcuate nucleus (ARC)-specific kisspeptin in the KNDy neuron that ARC kisspeptin is critical for estrous cyclicity in female mice and GnRH/LH pulsatility in both sexes. Our study reveals that ARC kisspeptin is essential for normal gametogenesis, and the loss of ARC kisspeptin results in significant hypogonadism, impacting fertility status. Our findings further confirm that normal puberty occurs despite a loss of ARC kisspeptin.

## Graphical abstract

**Figure:**
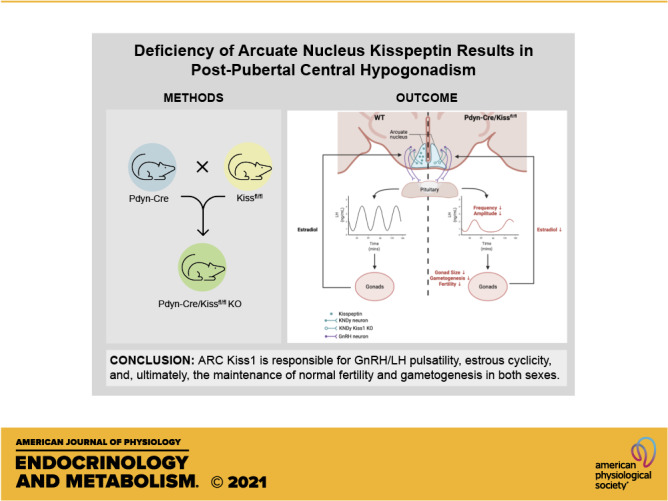

## INTRODUCTION

Kisspeptin, encoded by *Kiss1*, is a 54-amino acid polypeptide that binds to and activates the G-protein coupled receptor, kisspeptin receptor (Kiss1R encoded by *Kiss1r*). The critical role of kisspeptin in regulating normal pubertal onset and reproductive development came to light after the discovery of a family lacking *Kiss1r* and their clinical phenotype of idiopathic hypogonadotropic hypogonadism (1, 2). This finding was the first to suggest that kisspeptin has a major role in the hypothalamic-pituitary-gonadal (HPG) axis. Activation of central Kiss1R via kisspeptin administration in rats and monkeys elicits downstream effects on the HPG axis, including activation of the gonadotropin-releasing hormone (GnRH) neuron and GnRH secretion leading to the onset of puberty and normal reproductive cyclicity, providing evidence for kisspeptin’s role in regulating the reproductive axis (3–5). Several groups demonstrated the mechanism for kisspeptin action on GnRH secretion as a direct interaction between kisspeptin and Kiss1R expressed in GnRH neurons (6–8). Thus, the downstream effects of GnRH on stimulating the release of the gonadotropins, luteinizing hormone (LH), and follicle-stimulating hormone (FSH) are, in large part, regulated by hypothalamic kisspeptin neurons.

Hypothalamic kisspeptin is synthesized in two distinct and well-characterized nuclei—the arcuate nucleus (ARC) and the anteroventral periventricular nucleus (AVPV). AVPV kisspeptin neurons are thought to mediate estradiol-induced positive feedback and the preovulatory LH surge via the estrogen receptor (ER) α and are permissive for pubertal onset (9–11). ARC kisspeptin neurons are believed to be the neuroanatomical site for GnRH pulse generation (12–15), and mediate negative feedback of estradiol to regulate gonadotropin release and GnRH-mediated LH pulsatility (16). ARC kisspeptin neurons are distinguished by their coexpression of neurokinin B and dynorphin A. Due to the coexpression of these three neuropeptides, they are collectively referred to as KNDy neurons (17). Dynorphin and neurokinin B are thought to act as inhibitory and stimulatory influences, respectively, on arcuate kisspeptin expression to generate and coordinate pulsatile GnRH release in both males and females (18–20). Previous brain slice electrophysiology and in vivo optogenetic studies have demonstrated the role of the ARC kisspeptin neurons in the activation of the Kiss1R on GnRH neurons in the control of pulsatile GnRH synthesis. These studies suggest a G-protein coupled receptor-mediated mechanism of depolarization of GnRH neurons by ARC kisspeptin as well as the synchronized, potent, and unique ability of ARC kisspeptin in this process (21–23). Thus, hypothalamic kisspeptin plays an important role in the onset of puberty, ovulation, the maintenance of GnRH and LH pulse generation.

The literature currently lacks an exemplary in vivo model that fully dissects the independent functions of ARC and AVPV kisspeptin. Previous models targeting ARC kisspeptin have used toxin or viral-based ablation postnatally (24–26), or by examining a *Sox14* knockout (KO) mouse based on RNA-seq data suggesting that *Sox14* is an embryonic marker for ARC kisspeptin neurons (27) (models summarized in Table 1). All of these studies examine the effects of postnatal induction of ARC *Kiss1* deletion; or use toxin-based ablation or silencing approaches via stereotaxic surgery.

**Table 1.** Summary of findings in ARC Kiss1 KO rodent models

Phenotype	Pdyn-Cre/Kiss1^fl/fl^ KO	AAV1-DIO-GFP:TeTx	rAAV-Kisspeptin-AS	AAV-Cre + Kiss1^fl/fl^ KO
Deletion strategy	Cre-LoxP Embryonic deletion of *Pdyn*-expressing Kiss1 neurons	(TeTx) viral stereotaxic injection into ARC of adult Kiss1-Cre female mice	rAAV-Kisspeptin-antisense stereotaxic injection into ARC of adult female rats	AAV-Cre viral stereotaxic injection into ARC of adult Kiss1^fl/fl^ female rats
Body weight	(Pubertal) -	↑	NR	NR
Puberty onset (♀)	Normal	NA	NA	NA
Puberty onset (♂)	Normal	NA	NA	NA
Estrous cyclicity	Persistent Diestrus	Persistent Diestrus	Abnormal; Increased time between estrus	NR
LH pulsatility (♀)	↓	NR	(OVX + E_2_) ↓	(OVX + E_2_)↓
LH pulsatility (♂)	↓	NR	NR	NR
LH (♀)	Normal	↓	Normal	↓
LH (♂)	Normal	NR	NR	NR
FSH (♀)	Lower trend	NR	Normal	(Pituitary) ↓
FSH (♂)	↓	NR	NR	NR
Ovaries	Hypogonadal; ↓Weight	NR	No differences in corpora lutea/antral follicle counts	NR
Testes	Hypogonadal; ↓Weight	NR	NR	NR
Fertility (♀)	Infertile	NR	NR	NR
Fertility (♂)	Subfertile	NR	NR	NR
Serum E_2_ (♀)	↓	NR	Normal	NR
Reference	This manuscript	Padilla et al., 2019 (25)	Beale et al., 2014 (26)	Nagae et al., 2021 (24)

Comparison of parameters examined in the Pdyn-Cre/Kiss1^fl/fl^ KO with that of other published ARC Kiss1 knock-out rodent models. ↑, increase; ↓, decrease; -, no change; ARC, arcuate nucleus; FSH, follicle-stimulating hormone; KO, knockout; LH, luteinizing hormone; NA, not applicable, NR, not reported.

Thus, we generated a conditional ARC *Kiss1* knockout mouse model using embryonic targeting with Prodynorphin-IRES-Cre mouse line (Pdyn-Cre/Kiss1^fl/fl^ KO) (17, 18, 28) to discern ARC *Kiss1*-specific effects on the HPG axis. We hypothesize that in Pdyn-Cre/Kiss^fl/fl^ animals, the loss of ARC *Kiss1* will result in a loss of GnRH/LH pulsatile secretion, abnormal estrous cyclicity, and normal puberty onset. The results demonstrate the critical role of ARC kisspeptin in reproductive function. Pdyn-Cre/Kiss1^fl/fl^ KO females were in persistent diestrus and produced fewer LH pulses that interrupted folliculogenesis, caused hypogonadism, and infertility. Pdyn-Cre/Kiss1^fl/fl^ KO males had similarly disrupted LH pulsatility, defective spermatogenesis, hypogonadism, and variable fertility. Pubertal onset and development remained intact in males and females. These studies confirm the specific and critical role of ARC kisspeptin in mammalian reproduction.

## MATERIALS AND METHODS

### Animals

**Figure 1.**
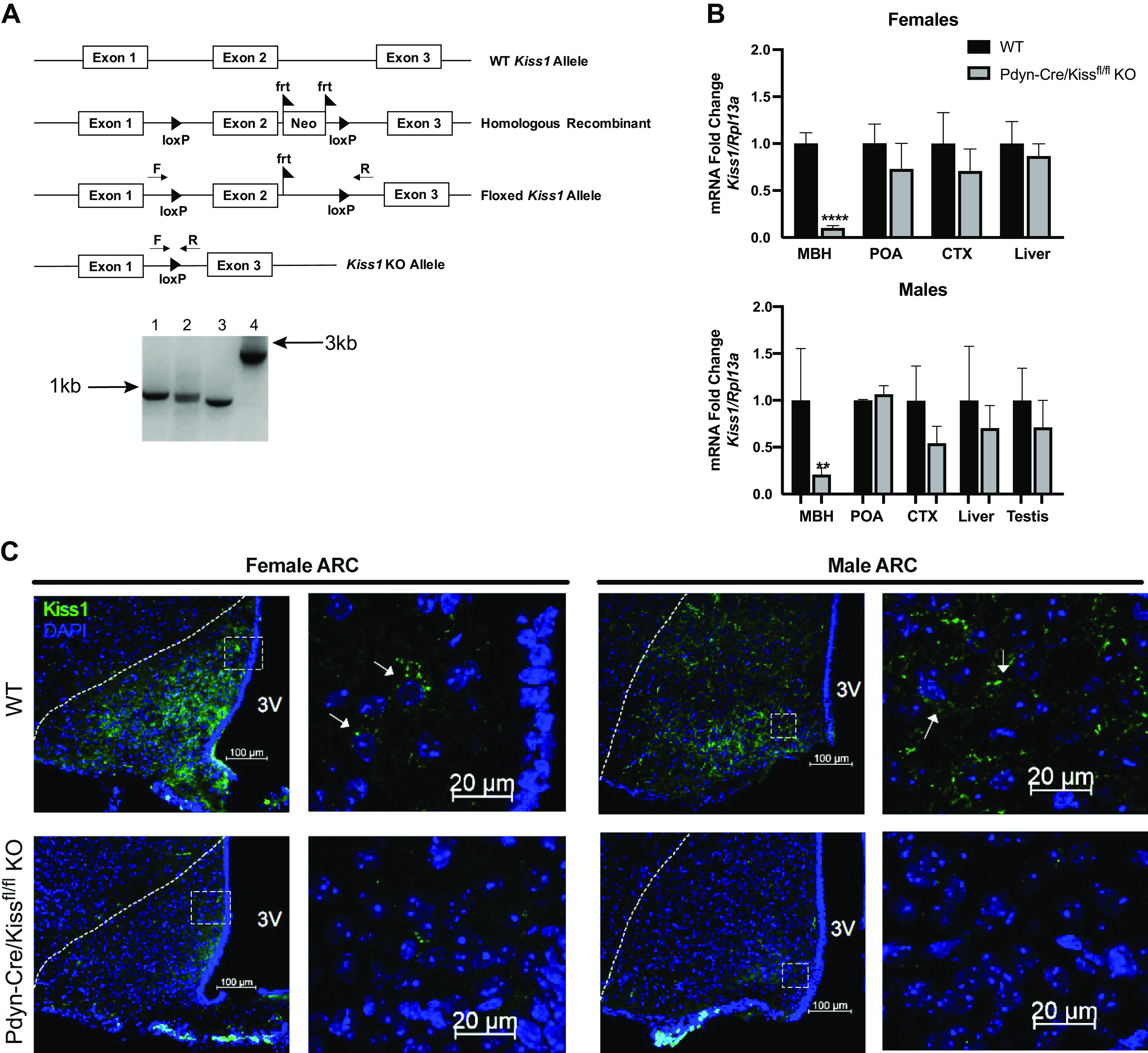
Validation of Pdyn-Cre/Kiss1^fl/fl^ knockout (KO) mouse. *A*: diagrams of *Kiss1* genetic constructs used to generate the Kiss1^flox^ mouse line in the 5′ to 3′ direction. The homologous recombinant expresses the two loxP sites around exon 2 of *Kiss1* and the neomycin cassette flanked with frt sites. Postmating a *Kiss1* homologous recombinant with a ROSA-FLP mouse, the neomycin cassette was excised, exhibited by the floxed *Kiss1* allele. Crossing homozygous floxed *Kiss1* mice with Pdyn-IRES-Cre mice resulted in *Kiss1* KO alleles in KNDy neurons. Genomic tail DNA was run through a PCR with forward and reverse *Kiss1* flox primers that bind outside of the loxP sites. The four allele combinations shown are as follows: *1*) flox/flox, *2*) flox/wild-type (WT), *3*) wild-type/wild-type, and *4*) the homologous recombinant including the neomycin cassette and frt sites prior to excision. *B*: relative mRNA expression of *Kiss1* was determined in the mediobasal hypothalamus (MBH), preoptic area (POA), neocortex (CTX), liver, and testes in Pdyn-Cre/Kiss1^fl/fl^ KO animals compared with WT controls within each tissue, within each sex. *Kiss1* mRNA in the MBH was significantly decreased in KO mice of both sexes (unpaired Student’s *t* test, ****P < 0.0001, **P < 0.01 KO vs. WT, (unpaired Student’s *t* test, WT *n* = 3–6, Pdyn-Cre/Kiss1^fl/fl^ KO *n* = 3–6). *Kiss1* mRNA in the POA was similar in KO mice of both sexes. 1–3 animals were removed as outliers after conducting Grubb’s outlier test. *C*: representative photomicrographs revealing kisspeptin peptide immunofluorescence in brain sections from the female arcuate nucleus (ARC) (*left,* four images) and male ARC (*right*, four images) of WT and Pdyn-Cre/Kiss1^fl/fl^ KO mice. In the ARC, a decrease in kisspeptin immunoreactivity in neurons in the Pdyn-Cre/Kiss1^fl/fl^ KO mice is seen. Magnification in the leftmost *top* and *bottom* panels is set at ×20, while magnification in the rightmost *top* and *bottom* panels is set at ×63. Scale bars are set at 100 µm for ×20 images, and 20 µm for ×63 images. Dotted lines in the ×20 image outline the boundary of the ARC. Boxes in the ×20 images denote the frame within which the ×63 images were taken. Arrows denote individual kisspeptin-ir positive cells. F, *Kiss1* flox forward primer; Neo, neomycin cassette; R, *Kiss1* flox reverse primer, 3V, third ventricle.

Prodynorphin-IRES-Cre mice, established by Dr. Bradford Lowell at Harvard University (28), were purchased from The Jackson Laboratory (Stock No. 027958) and crossed to a *Kiss1* floxed (Kiss1^fl/fl^) mouse line generated in our laboratory. Kiss1^fl/fl^ mice were generated by using a bacterial artificial chromosome (BAC) clone DNA construct containing the entire coding sequence of all known *Kiss1* splice variants and the coding and regulatory regions of *Golt1a* (Clone BMQ-203-m22, Source Bioscience). The coding sequence for kisspeptin is located in exons 2 and 3 for all transcript variants. Recombineering technology (29, 30) was used to insert LoxP sites flanking exon 2, which includes the translational start site for *Kiss1*. Cre-mediated excision of exon 2 results in a frameshift mutation in exon 3 to produce a truncated protein product and complete absence of the kisspeptin protein. This targeted fragment was electroporated into embryonic stem cells of 129SV/J mice by the Johns Hopkins University ES Cell Targeting Core Laboratory to target *Kiss1* using homologous recombination. After three successful recombination events were identified, they were injected into blastocysts at the Johns Hopkins Transgenic Core. Several high-grade chimeric mice were chosen to breed and demonstrated germline transmission of the modified allele. Mating the F1 mice with a ROSA-flippase mouse resulted in excision of the FRT-flanked neomycin cassette (Fig. 1*A*), and F2 generated Kiss1^fl/fl^ mice. The knockout (KO) mice are identified as Pdyn-Cre^+/−^ and Kiss1^fl/fl^, verified through PCR genotyping (Supplemental Table S1; all Supplemental material is available at https://figshare.com/s/0019f2137f93b6f16a28, Fig. 1*A*). Kisspeptin neurons begin to develop in the ARC at E13.5, significantly increasing in number by E16.5 (31), whereas the number of prodynorphin-expression neurons in the developing hypothalamus are significantly increased by E18.5 (©2008 Allen Institute for Brain Science. Allen Developing Mouse Brain Atlas. Available from https://developingmouse.brain-map.org/experiment/show/100057203)—thus, the deletion of *Kiss1* is mediated by Pdyn-IRES-Cre in utero. Mice used in this study were maintained on a mixed C57B6/J and 129SV/J background. Mice were genotyped using tail DNA and primer sequences for Pdyn-Cre [mutant band size: 110 base pairs, wild-type (WT) band size: 425 base pairs] and *Kiss1* flox (flox band size: 1,368 base pairs, wild-type band size: 1,266 base pairs) (Supplemental Table S1). Animals were housed in the Child Health Institute of NJ (CHINJ) vivarium, maintained on a 12:12 light/dark cycle (lights ON at 6:00 AM) and were fed ad libitum, with free access to water. Control mice were identified as Kiss1^fl/fl^ or Kiss1^fl/wt^. All experimental procedures adhered to the guidelines provided in the National Institutes of Health Guide for the Care and Use of Laboratory Animals and were approved by the Institutional Animal Care and Use Committee (IACUC) at Johns Hopkins University and Rutgers University. Kiss^fl/fl^ transgenic mouse generation was completed at Johns Hopkins University, while all further experiments were performed at Rutgers University.

### Quantitative Reverse Transcription PCR

Tissue from adult mice aged 2–6 mo was freshly dissected and immediately frozen on dry ice, then stored at −80°C until RNA extraction was performed. The mediobasal hypothalamus (MBH) and preoptic area (POA) were collected using a mouse brain matrix (Roboz) following a blocked-section collection method (32). RNA was homogenized with the Qiagen TissueRuptor II handheld rotor-stator homogenizer and subsequently extracted from tissue using the TRIzol RNA isolation protocol by Thermo Fisher Scientific. RNA was then reverse transcribed into cDNA using iScript cDNA Synthesis Kit (Bio-Rad) according to manufacturer’s protocol. cDNA was diluted 1:1 with Molecular-Grade Water (Sigma-Aldrich) and genes amplified using Universal SYBR Green Master Mix (Bio-Rad) in accordance with their protocol. Values were normalized to the wild-type tissue for each tissue type, within each sex. The primers used for gene amplification are shown in Supplemental Table S1. Outliers were identified using Grubb’s outlier test and excluded from analysis.

### Perfusion and Brain Immunofluorescence

Mice were anesthetized with a cocktail mix of ketamine/xylazine (100 and 10 mg/kg of body weight, respectively) and then underwent intracardial perfusion with 4% paraformaldehyde (PFA, pH 7.4) made in 0.1 M PBS. Brains were then extracted, postfixed overnight in 4% PFA at 4°C, and then placed in 30% sucrose until fully infiltrated. Brains were then frozen on dry ice and stored at −80°C until sectioning. Coronal brain sections were obtained at 30-µm thickness using a cryostat, starting from the rostral preoptic area (Bregma 0.62 mm) to the caudal extent of the arcuate nucleus (Bregma −2.80 mm) according to Franklin and Paxinos (33). Sections were serially collected in cryoprotectant and stored at −20°C until staining.

Free-floating brain sections were washed four times in 1× PBS for 10 min each, then blocked for 1 h at room temperature in 5% normal goat or horse serum and 0.3% Triton X-100 in 1× PBS. Sections were then incubated in a 1:10,000 dilution of primary antibody (AC No. 566, Rabbit Polyclonal anti-kisspeptin-10, developed by Dr. Alain Caraty, RRID: AB_2314709), in the same blocking solution for 1 h at room temperature, and then for 48 h at 4°C (34). After washing in 1× PBS, sections were then incubated in Alexa 488 goat or donkey anti-rabbit secondary antibody (1:500 dilution; Invitrogen/Thermo Fisher), made in 0.3% Triton X-100 in 1× PBS, for 3 h at room temperature and protected from light. Sections were then washed four times in 1× PBS and mounted onto Superfrost glass slides. Finally, slides were coverslipped with DAPI Fluoromount-G (Southern Biotech) and left to dry until imaging. Kisspeptin-ir in the ARC was analyzed in brain sections that encompassed Bregma −1.22 mm to −2.46 mm from at least three sections per animal in female WT (*n* = 4) and Pdyn-Cre/Kiss1^fl/fl^ KO (*n* = 3), and male WT (*n* = 3) and Pdyn-Cre/Kiss1^fl/fl^ KO (*n* = 3).

### Microscopy and Imaging

Images were acquired with a Zeiss 700 Laser Scanning Microscope (LSM700) using a Plan-Apochromat ×20 objective and ×63 water-immersion objective and processed with Zen 2.3 software (Zeiss, Germany). Each channel visualized was scanned individually to prevent crosstalk between channels and fluorophores.

### Pubertal Development and Growth

External markers of pubertal onset were assessed in female and male mice of both genotypes for comparison. In females, pubertal onset was determined as the presence of vaginal opening (VO) and was assessed after daily clinical observations starting from the day of weaning (21 days of age). In males, pubertal onset was defined as the day that balanopreputial separation was fully achieved, and this was assessed daily starting at weaning age. Preputial separation was assessed by gentle manual retraction of the prepuce (35). Anogenital distance in males, defined here as the distance from the anus to the genital tubercle at 25 days of age, was measured as an external sign of androgen-sensitive growth (36–38). Body weight in females and males of both genotypes was measured on a tared laboratory scale every two days starting at weaning age (21 days) through 49 days of age.

### Assessment of Estrous Cyclicity

Starting on the day of VO, female mice underwent daily vaginal lavage with 0.9% saline solution for observation of vaginal cytology to determine the stages of the estrous cycle. This method was used to determine the day of first estrus to indicate their first ovulatory event. Estrous cyclicity was determined starting at 2 mo of age, when reproductive maturity was reached. The stage of the estrous cycle was determined by observing vaginal epithelial cytology and assessing the proportion of leukocytes, nucleated epithelial cells, and cornified cells present in the collected saline samples under a bright-field microscope (39).

### Gonadal Histology

Ovaries were freshly dissected and fixed in 4% PFA for at least 24 h before transferring to 70% EtOH. Testes were freshly dissected and fixed in Bouin’s solution (Sigma-Aldrich) for at least 24 h before transferring to 70% EtOH. Fixed gonads were kept at 4°C before paraffin embedding by Rutgers University’s Pathology Core. Mouse ovaries and testes were sectioned cross-sectionally at 5 µm thickness and stained using histological hematoxylin and eosin staining. To determine gonad weights, both sets of ovaries and both sets of testes, freshly dissected, were cleaned of gonadal white adipose tissue and weighed on a tared laboratory scale, before fixation.

To determine corpora lutea counts, ovaries were sectioned at 5 µm in thickness and sections collected at 200-µm increments (40) throughout the entire ovary and stained using histological hematoxylin and eosin staining. The number of corpora lutea in each section was estimated and summed for all sections in each ovary.

### Measurement of Gonadal and Uterine Size

Approximate total cross-sectional area for ovaries and testes were conducted using FIJI software (ImageJ, NIH) and the provided threshold analysis function. The total area in microns was calibrated to the scale bar provided in each scanned image. Total area for the uterine layers (the myometrium and endometrium) was conducted using Aperio ImageScope software and the provided pen and annotation measurement tools.

### Tail-Tip Blood Collection and Ultrasensitive Mouse LH Assay

Female and male mice (2–3 mo old) underwent serial tail-tip blood collection between 1000 h and 1300 h. Female mice underwent blood collection on the day of diestrus. Mice were acclimated by daily handling and placing in a mouse restrainer before the experiment. Tail-tip blood (6 µL) was collected from mice, while inside a mouse restrainer, and directly pipetted into 54 µL of assay buffer (1:10 dilution) prepared according to the protocol provided by the University of Virginia Center for Research in Reproduction Ligand Assay and Analysis Core. Blood was collected and immediately frozen on dry ice every 6 min for 3 h, then stored at −80°C until analysis at the UVA Ligand Assay Core. The functional sensitivity, defined as the lowest concentration that demonstrates accuracy within 20% of expected values, is 0.016 ng/mL. Intra-assay coefficient of variance (CV) is 2.2%. Interassay CVs are 7.3% [low quality control (QC), 0.13 ng/mL], 5.0% (medium QC, 0.8 ng/mL), and 6.5% (high QC, 2.3 ng/mL) (41).

### LH Pulsatility Analysis

LH pulses were determined based upon the following criteria to maximize stringency with gonad-intact animals: *1*) peaks must have at least a 30% increase from the previous 1 or 2 LH values, *2*) peaks must be followed by a 10% decrease in the next 1 or 2 LH values, and *3*) pulse amplitude (LH increase from nadir to peak) must be greater or equal to 0.48 ng/mL or three times the assay’s lower detection limit (42–44). LH pulsatility dynamics were assessed by calculating the following parameters: *1*) Pulse frequency, calculated as the number of pulses in the 3-h collection period, *2*) Pulse amplitude, calculated as the difference between the pulse peak and the preceding nadir, and *3*) Mean LH, calculated by averaging all LH values in the 3-h collection period for each animal.

### Fertility Study

Starting between 2–3 mo of age, wild-type (WT) and knockout (KO) virgin female mice were individually paired with a proven stud WT male for 90 days. Mice were checked daily for plugs and pups. The number of litters, the number of pups per litter, and the day-of-birth were recorded. After the pups were born, they were immediately euthanized, and the dam was placed with the stud male for the remainder of the 90-day period.

Starting between 2–3 mo of age, WT and KO virgin male mice were paired with a proven fertile WT female for a minimum of 10 days, before separating and pairing the male with a different proven fertile WT female. The females were observed for pregnancy and full-term birth. The number of litters, the number of pups per litter, and the day-of-birth were recorded. The reproductive success rate of the females and males was determined by the number of successful (litter-bearing) matings divided by the number of total matings.

### FSH Multiplex Assay

Serum from whole blood, collected during euthanization, was collected using the UVA Center for Research in Reproduction Ligand Assay and Analysis Core’s protocol. Samples were allowed to clot for 90 min before centrifugation at 2,000 *g* for 15 min at room temperature. After centrifugation, serum was carefully pipetted into separate tubes and stored at 4°C before shipment for analysis. The mouse FSH Multiplex assay (Millipore Pituitary Panel Multiplex kit) was performed by the UVA Center for Research in Reproduction Ligand Assay and Analysis Core. The reportable range for the multiplex assay is 0.24–30 ng/mL. Outside the reportable range is classified as %CV > 20.

### Serum Estradiol LC-MS Analysis

The serum used in this assay was collected from diestrous female mice at the time of euthanization. Estradiol measurement was conducted as previously described (45, 46). Briefly, internal standard was added to pooled serum samples (257–350 μL), which were extracted twice using methyl *tert*-butyl ether followed by dichloromethane. Samples were derivatized using dansyl chloride and then analyzed by LC-MS/MS (Sciex QTRAP 5500). Individual calibration curves were constructed for each analyte with at least 8 points. The linearity was *r* > 0.9990 and the curve fit was linear with 1/*x* weighting. None of the compounds of interest were detected in blank or double blank samples. The interassay coefficient of variation was determined by a pool of human serum and ranged from 8.62% to 11.88%. The limit of detection for this assay is 1.22 pg. The pg/mL value from the samples was adjusted based on the volume of serum used.

### Statistical Analyses

All statistical analyses were done using GraphPad Prism Version 8. Statistical analysis comprised of the parametric, unpaired Student’s *t* test to compare wild-type and knockout groups. Statistical significance was defined as *P* < 0.05. Grubb’s outlier test was conducted, and all identified outliers (significance <0.05) were excluded from analysis. All error bars indicate standard error of the mean (SEM).

## RESULTS

### Deletion of *Kiss1* in the ARC

Although coexpression of kisspeptin and prodynorphin is not reported in cell populations other than arcuate KNDy neurons, we validated the genetic specificity and accuracy of the knockout model. In both sexes, qRT-PCR analysis (Fig. 1*B*) conducted on a tissue panel demonstrated a significant reduction in *Kiss1* mRNA expression in the mediobasal hypothalamus (MBH), which contains the ARC kisspeptin neurons, in Pdyn-Cre/Kiss1^fl/fl^ KO mice when compared with wild-type controls (WT) of the same sex (unpaired Student’s *t* test, Females: ****P < 0.0001 KO vs. WT; Males: **P < 0.01 KO vs. WT). Importantly, *Kiss1* expression in the preoptic area (POA), where AVPV kisspeptin neurons reside, was equivalent between Pdyn-Cre/Kiss1^fl/fl^ KO animals and controls (unpaired Student’s *t* test, Females: *P* = 0.6445 KO vs. WT; Males: *P* = 0.9839 KO vs. WT). Thus, AVPV *Kiss1* expression remains unchanged and supports the specificity of our knockout model. To further validate our qRT-PCR data, we evaluated kisspeptin protein expression in brain sections using immunofluorescence staining. We assessed kisspeptin-immunoreactivity (ir) in the ARC in brain sections between Bregma −1.22 mm and −2.46 mm (*n* = 3–4 animals per genotype, per sex) both at ×20 and ×63 magnifications. WT brain sections demonstrated normal kisspeptin-ir patterns in both ARC and AVPV; Pdyn-Cre/Kiss1^fl/fl^ KO mice of both sexes had a loss of kisspeptin-ir specifically in the ARC, whereas kisspeptin-ir in the AVPV of female and male KO mice were comparable with sex-matched controls (Fig. 1*C*, Supplemental Fig. S1). The decrease in *Kiss1* expression in both female and male Pdyn-Cre/Kiss1^fl/fl^ KO MBH combined with the complete loss of kisspeptin immunoreactivity in the ARC of Pdyn-Cre/Kiss1^fl/fl^ KO female and male mice demonstrate the specificity of the Cre-mediated deletion of ARC kisspeptin.

### Arcuate *Kiss1* Is Not Required for Pubertal Onset in Mice

**Figure 2.**
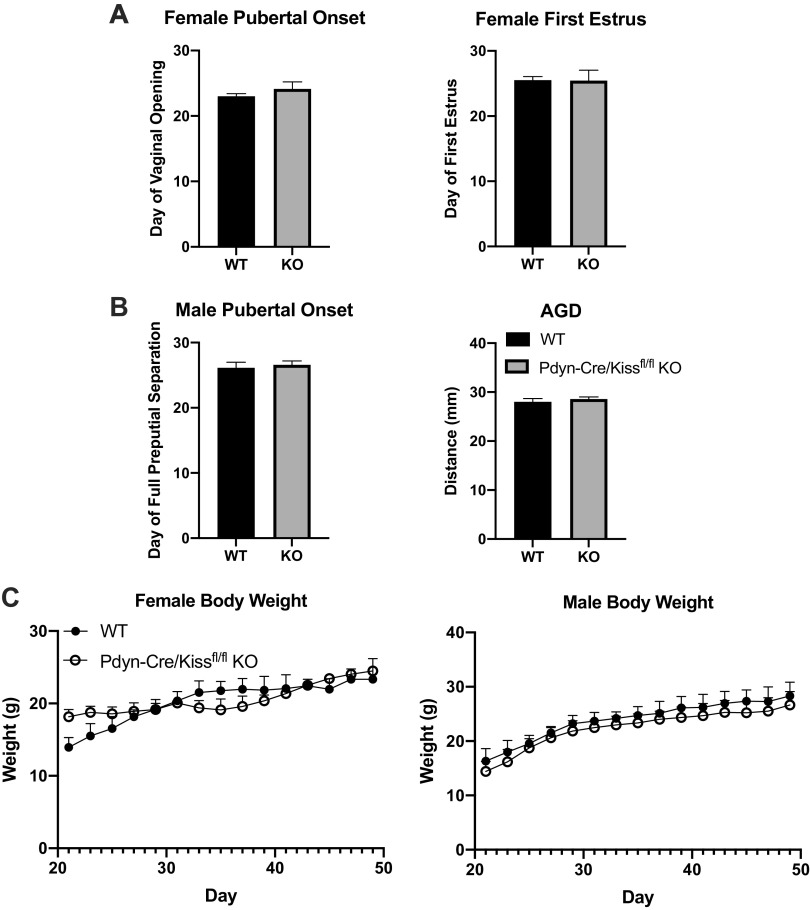
Puberty onset and body weight of PdynCre/Kiss1^fl/fl^ knockout (KO) mice. *A*: pubertal onset for female mice was assessed by daily observation after weaning (age 21 days) to the day of vaginal opening [unpaired Student’s *t* test, wild type (WT) *n* = 11, Pdyn-Cre/Kiss1^fl/fl^ KO, *n* = 7]. After vaginal opening, vaginal cytology was assessed by daily saline lavage to determine the date of first estrus (unpaired Student’s *t* test, WT *n* = 6, Pdyn-Cre/Kiss1^fl/fl^ KO *n* = 7). *B*: pubertal onset for male mice was assessed by daily observation after weaning (age 21 days) until the day of full balanopreputial separation (unpaired Student’s *t* test, WT *n* = 8, Pdyn-Cre/Kiss1^fl/fl^ KO *n* = 12). Anogenital distance (AGD) was measured at 25 days of age for each cohort and shown to be similar between genotypes (unpaired Student’s *t* test, WT *n* = 10, Pdyn-Cre/Kiss1^fl/fl^ KO *n* = 12). *C*: body weight gain throughout the duration of pubertal onset. Mice were weighed every other day from weaning until 49 days of age. Body weights for both sexes remained similar throughout the duration of puberty (Bonferroni’s multiple comparisons test, Females: WT *n* = 4, Pdyn-Cre/Kiss1^fl/fl^ KO *n* = 3, Males: WT *n* = 6, Pdyn-Cre/Kiss1^fl/fl^ KO *n* = 9).

We evaluated external parameters indicating the onset of puberty in male and female Pdyn-Cre/Kiss1^fl/fl^ KO mice (Fig. 2, *A* and *B*). For females, the day of vaginal opening was evaluated as an indicator of puberty onset, whereas in males we evaluated the day of full preputial separation. In both sexes, there was no significant difference between WT and Pdyn-Cre/Kiss1^fl/fl^ KO groups in these indicators of pubertal onset (unpaired Student’s *t* test, Females: *P* = 0.2823 KO vs. WT; Males: *P* = 0.6548 KO vs. WT). In addition, vaginal cytology was assessed in females starting from the day of vaginal opening to monitor for cornified cells signifying the day of first estrus, which is an indicator of their first ovulatory event. The day of first estrus was not significantly different in Pdyn-Cre/Kiss1^fl/fl^ KO females compared with WT females (unpaired Student’s *t* test, *P* = 0.9693). In pubertal males, anogenital distance (AGD) was used as an indicator of testosterone responsiveness; AGD in Pdyn-Cre/Kiss1^fl/fl^ KO males was not significantly different from that of the WT group (unpaired Student’s *t* test, *P* = 0.4514). Body weight gain during puberty was not significantly different for mice of both sexes between genotypes at any timepoint (Bonferroni’s multiple comparisons test) (Fig. 2*C*).

### Adult Pdyn-Cre/Kiss1^fl/fl^ KO Females Exhibit Abnormally Long Periods in Diestrus

**Figure 3.**
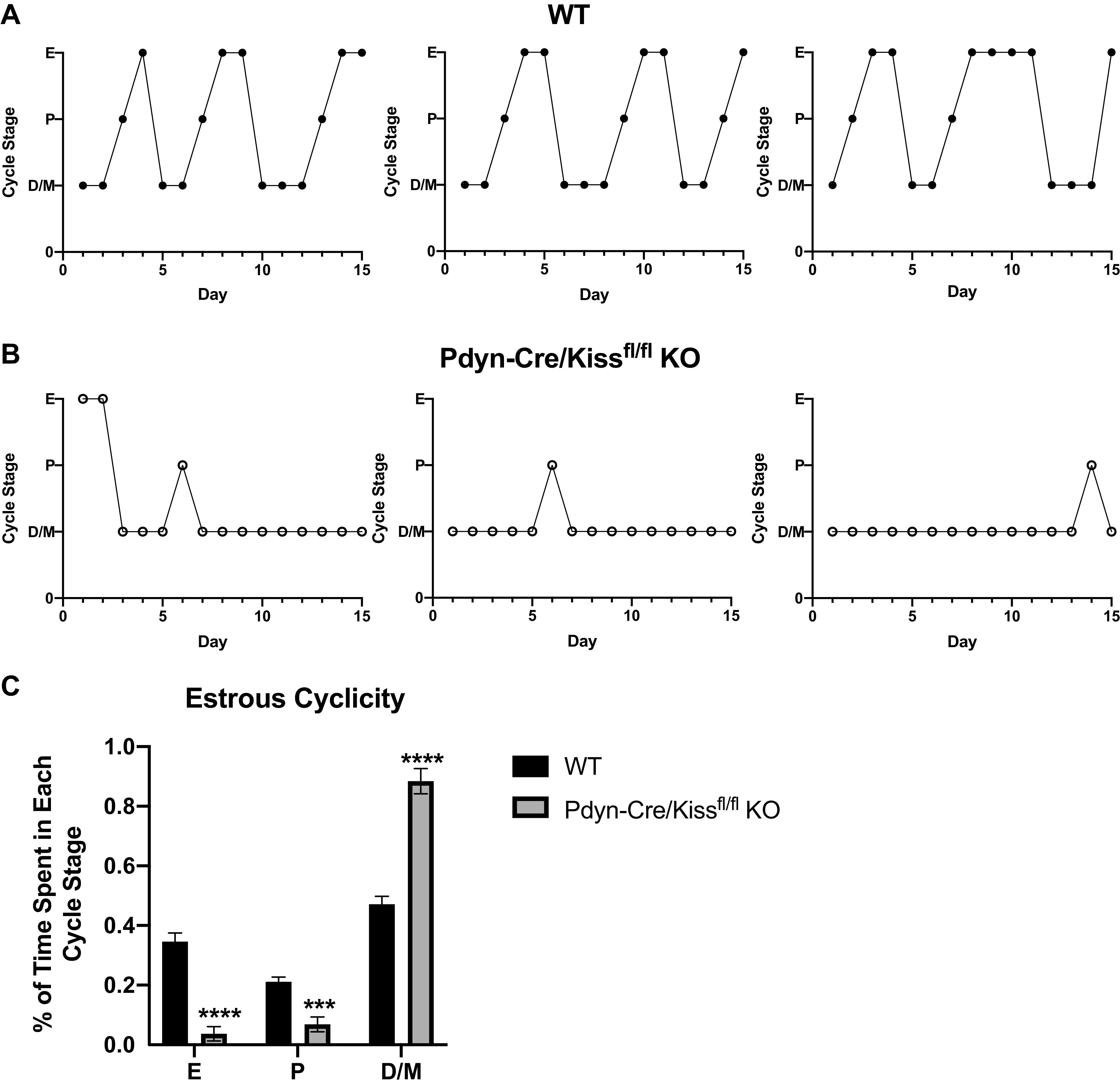
Estrous cyclicity in Pdyn-Cre/Kiss1^fl/fl^ knockout (KO) mice. At 2 mo of age, vaginal cytology was collected and assessed for 15 consecutive days. *A*: representative wild-type (WT) female estrous cycles. *B*: representative Pdyn-Cre/Kiss1^fl/fl^ KO female estrous cycles. *C*: percent of time mice spent in each estrous cycle stage. Pdyn-Cre/Kiss1^fl/fl^ KO mice demonstrated persistent diestrus compared with regular cycles observed in WT mice. Pdyn-Cre/Kiss1^fl/fl^ KO females spent significantly less time in estrus and proestrus and more time in diestrus/metestrus (unpaired Student’s *t* test, ****P < 0.0001, ***P < 0.001 Pdyn-Cre/Kiss1^fl/fl^ KO vs. WT, WT *n* = 8, Pdyn-Cre/Kiss1^fl/fl^ KO *n* = 7). D/M, diestrus/metestrus; E, estrus; P, proestrus.

Estrous cyclicity was assessed by daily examination of vaginal cytology starting at reproductive maturity at 2 mo of age (Fig. 3, *A* and *B*). As expected, WT females demonstrated regular 4- to 5-day estrous cycles, exhibiting all stages of the cycle—proestrus, estrus, and metestrus/diestrus. However, age-matched Pdyn-Cre/Kiss1^fl/fl^ KO females demonstrated persistent metestrus/diestrus with occasional proestrus and estrus events (Fig. 3*C*, unpaired Student’s *t* test, ****P < 0.0001, ***P < 0.001 KO vs. WT). Our summarized findings between the WT and Pdyn-Cre/Kiss1^fl/fl^ KO estrous cycles demonstrate that Pdyn-Cre/Kiss1^fl/fl^ KO mice spend significantly less time in estrus and proestrus, while spending more time in metestrus/diestrus.

### Adult Pdyn-Cre/Kiss1^fl/fl^ KO Mice Have Decreased LH Pulse Frequency

**Figure 4.**
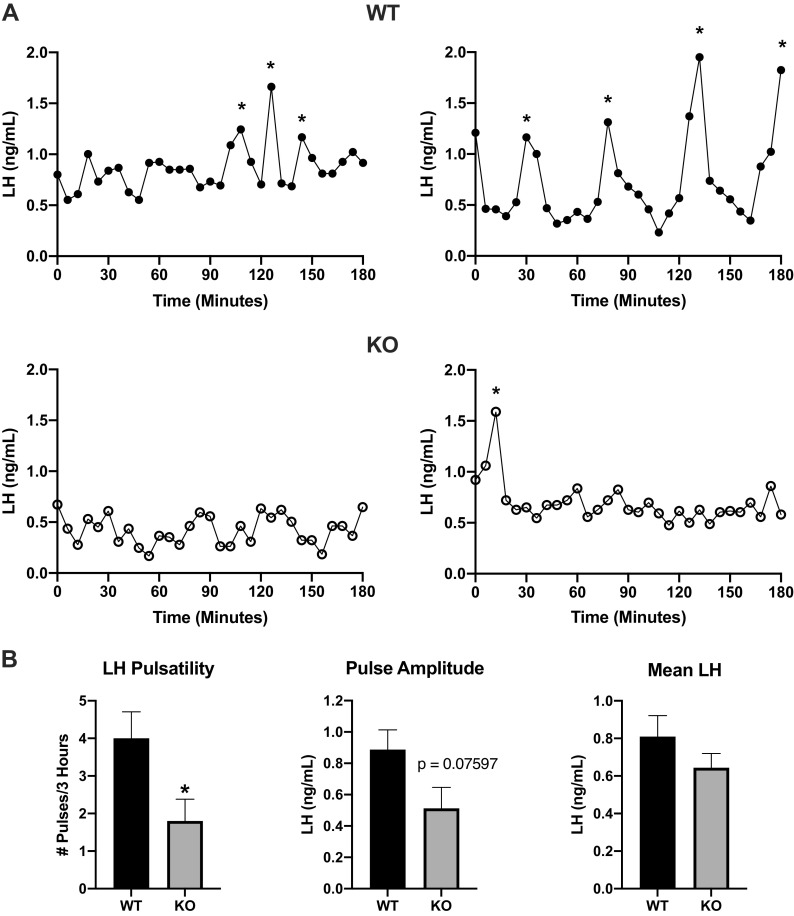
Luteinizing hormone (LH) pulsatility in female Pdyn-Cre/Kiss1^fl/fl^ knockout (KO) mice. *A*: representative profiles of LH pulsatile release in ovary-intact females. LH for pulse analysis was collected using serial tail-tip blood collections every 6 min for 3 h beginning between 10 and 11 AM on the day of diestrus. *Identified pulses. *B*: parameters of LH pulsatility included pulse frequency, pulse amplitude, and mean LH. Pulses are significantly less frequent in Pdyn-Cre/Kiss1^fl/fl^ KO mice compared with wild-type (WT) mice (unpaired Student’s *t* test, *P < 0.05 KO vs. WT, WT *n* = 5, Pdyn-Cre/Kiss1^fl/fl^ KO *n* = 5) while pulse amplitude (WT *n* = 5, Pdyn-Cre/Kiss1^fl/fl^ KO *n* = 5) was reduced by 42% (not statistically significant) and mean LH remained similar between genotypes.

**Figure 5.**
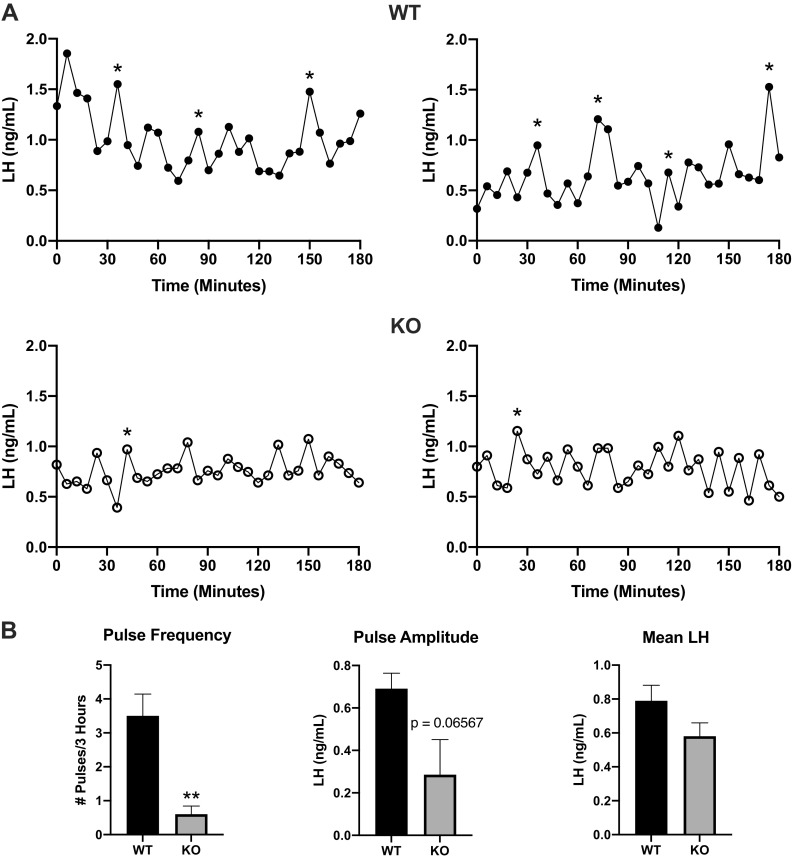
Luteinizing hormone (LH) pulsatility in male Pdyn-Cre/Kiss1^fl/fl^ knockout (KO) mice. *A*: representative profiles of LH pulsatile release in testes-intact males. LH for pulse analysis was collected using serial tail-tip blood collections every 6 min for 3 h starting between 10 and 11 AM. *Identified pulses. *B*: parameters of LH pulsatility were pulse frequency, pulse amplitude, and mean LH. Pulses are significantly less frequent in Pdyn-Cre/Kiss1^fl/fl^ KO mice compared to wild-type (WT) mice (unpaired Student’s *t* test, **P < 0.01 KO vs. WT, WT *n* = 4, Pdyn-Cre/Kiss1^fl/fl^ KO *n* = 5), while pulse amplitude (WT *n* = 4, Pdyn-Cre/Kiss1^fl/fl^ KO *n* = 5) was reduced by 59% (not statistically significant) and mean LH remained similar between genotypes.

KNDy neurons in the ARC are thought to be the site of GnRH pulse generation (12–15). Hence, we sought to investigate how the loss of ARC kisspeptin (the major secretagogue for GnRH) affected the dynamic secretion of LH, as a proxy for GnRH secretion. Serial tail-tip blood samples were collected on the morning of diestrus (Fig. 4) and Pdyn-Cre/Kiss1^fl/fl^ KO females demonstrated a significant decrease in pulse frequency during the 3-h testing interval compared with that of WT females (unpaired Student’s *t* test, *P < 0.05 KO vs. WT). Pulse amplitude (unpaired Student’s *t* test *P* = 0.07597 KO vs. WT) had a 42% reduction in Pdyn-Cre/Kiss1^fl/fl^ KO females. Mean LH (unpaired Student’s *t* test, *P* = 0.2527 KO vs. WT) was similar between WT and Pdyn-Cre/Kiss1^fl/fl^ KO females (Fig. 4*B*). We also assessed LH pulsatility in adult Pdyn-Cre/Kiss1^fl/fl^ KO males (Fig. 5). Similar to our findings in Pdyn-Cre/Kiss1^fl/fl^ KO females, LH pulse frequency in Pdyn-Cre/Kiss1^fl/fl^ KO males was significantly decreased than compared with WT males (unpaired Student’s *t* test, **P < 0.01 KO vs. WT). Similarly, there was a 59% reduction in pulse amplitude (unpaired Student’s *t* test, *P* = 0.06567 KO vs. WT) Pdyn-Cre/Kiss1^fl/fl^ KO males, whereas mean LH (unpaired Student’s *t* test, *P* = 0.1282, KO vs. WT) was similar between genotypes in males.

### Adult Pdyn-Cre/Kiss1^fl/fl^ KO Females Exhibit Signs of Arrested Folliculogenesis

**Figure 6.**
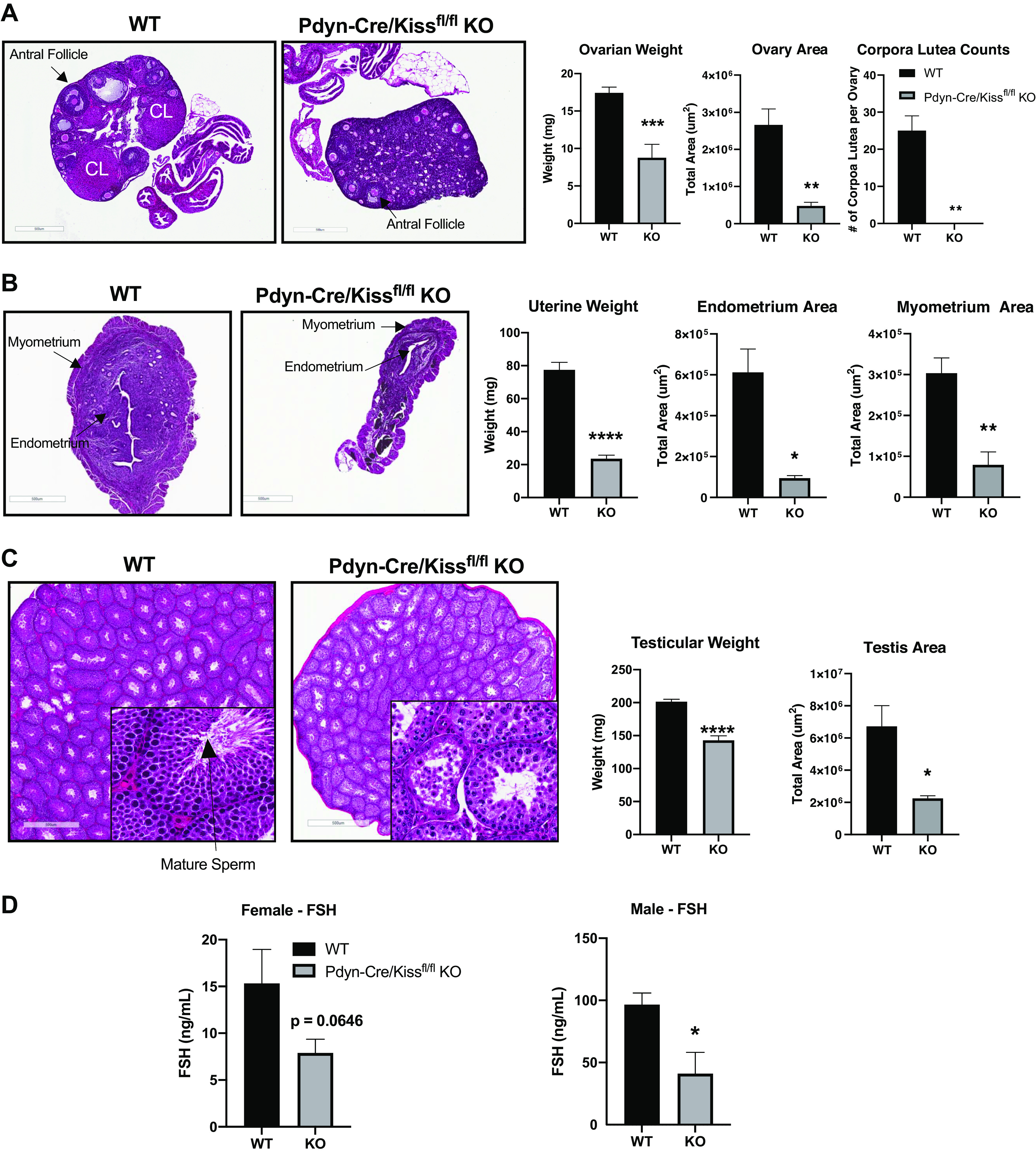
Gonadal/uterine assessment of Pdyn-Cre/Kiss1^fl/fl^ knockout (KO) mice. Age-matched adult mice ranged from 2 to 6 mo old. Each full set of gonads was weighed together. *A*–*C*: 5-μm sections of the ovary, uterus, and testis were obtained and stained with hematoxylin and eosin (H&E). *A*: Pdyn-Cre/Kiss1^fl/fl^ KO ovaries have more early-stage follicles, fewer antral follicles, and no corpora lutea [unpaired Student’s *t* test, **P < 0.01 KO vs. wild type (WT), WT *n* = 3, Pdyn-Cre/Kiss1^fl/fl^ KO *n* = 3]. Corpora lutea were estimated from the entire ovary within each 5 μm section in 200 μm increments. Weight and area of ovaries were significantly decreased in Pdyn-Cre/Kiss1^fl/fl^ KO mice (unpaired Student’s *t* test, ***P < 0.001, **P < 0.01 KO vs. WT, WT *n* = 8, Pdyn-Cre/Kiss1^fl/fl^ KO *n* = 5). *B*: Pdyn-Cre/Kiss1^fl/fl^ KO uteri were significantly lighter and thinner than WT uteri (unpaired Student’s *t* test, *P < 0.05, **P < 0.01, ****P < 0.0001 KO vs. WT, WT *n* = 7, Pdyn-Cre/Kiss1^fl/fl^ KO *n* = 3 for area, *n* = 4 for weight). *C*: Pdyn-Cre/Kiss1^fl/fl^ KO testes lacked mature spermatids and weighed significantly less when compared with WT testes (unpaired Student’s *t* test, *P < 0.05, ****P < 0.0001 KO vs. WT, WT *n* = 7, Pdyn-Cre/Kiss1^fl/fl^ KO *n* = 7). *D*: follicle-stimulating hormone (FSH) levels were measured in serum collected during terminal tissue collection. Serum FSH was found to be similar between WT (*n* = 10) and Pdyn-Cre/Kiss1^fl/fl^ KO (*n* = 9) diestrous females (unpaired Student’s *t* test KO vs. WT, FSH: *P* = 0.0646) and significantly decreased in Pdyn-Cre/Kiss1^fl/fl^ KO males (unpaired Student’s *t* test, *P < 0.05 KO vs. WT, WT *n* = 7, Pdyn-Cre/Kiss1^fl/fl^ KO *n* = 7). CL, corpus luteum.

To determine whether the irregular estrous cyclicity in Pdyn-Cre/Kiss1^fl/fl^ KO females was related to gonadal development, we examined ovarian morphology. Hematoxylin and eosin (H&E) staining of the innermost sections of the WT ovary revealed evidence of each stage of progressive folliculogenesis including primary/secondary follicles, antral follicles and corpora lutea. Qualitative analysis of Pdyn-Cre/Kiss1^fl/fl^ KO ovaries, however, revealed a larger proportion of early-stage follicles and antral follicles, but no corpora lutea. Ovarian weight and total cross-sectional area of the ovaries in Pdyn-Cre/Kiss1^fl/fl^ KO females was significantly lower when compared with WT ovaries (unpaired Student’s *t* test, weight: ***P < 0.001, area: **P < 0.01 KO vs. WT). Corpora lutea counts from whole ovaries showed a complete absence of corpora lutea from Pdyn-Cre/Kiss1^fl/fl^ KO ovaries when compared with WT ovaries (unpaired Student’s *t* test, **P < 0.01 KO vs. WT) (Fig. 6*A*). Uterine weight and total cross-sectional area of the endometrium and myometrium layers in Pdyn-Cre/Kiss1^fl/fl^ KO females was also significantly lower compared with WT uteri (unpaired Student’s *t* test, weight: ****P < 0.0001, endometrium area: *P < 0.05, myometrium area: **P < 0.01 KO vs. WT) (Fig. 6*B*). Furthermore, Pdyn-Cre/Kiss1^fl/fl^ KO males had significantly lower wet testicular weight compared with WT testes. The observed spermatogenesis in Pdyn-Cre/Kiss1^fl/fl^ KO males varied where most lacked mature sperm, while others maintained proper spermatogenesis comparable with that of WT males. Similar to that of the ovaries, the total cross-sectional area of the Pdyn-Cre/Kiss1^fl/fl^ KO testes was significantly less than the total area of WT testes (unpaired Student’s *t* test, weight: ****P < 0.0001, area: *P < 0.05 KO vs. WT) (Fig. 6*C*).

### Serum FSH Levels Were Decreased in the Pdyn-Cre/Kiss1^fl/fl^ KO

FSH multiplex assay conducted on serum from WT and Pdyn-Cre/Kiss1^fl/fl^ KO mice revealed that FSH was significantly decreased in Pdyn-Cre/Kiss1^fl/fl^ KO males. Although serum FSH levels were lower in Pdyn-Cre/Kiss1^fl/fl^ KO females compared with WT females, this reduction did not reach statistical significance (*P* = 0.0646). Serum for both Pdyn-Cre/Kiss1^fl/fl^ KO males and females reflected the mean LH values seen in the LH pulsatility studies (Fig. 6*D*).

### Pdyn-Cre/Kiss1^fl/fl^ KO Females Are Infertile

**Figure 7.**
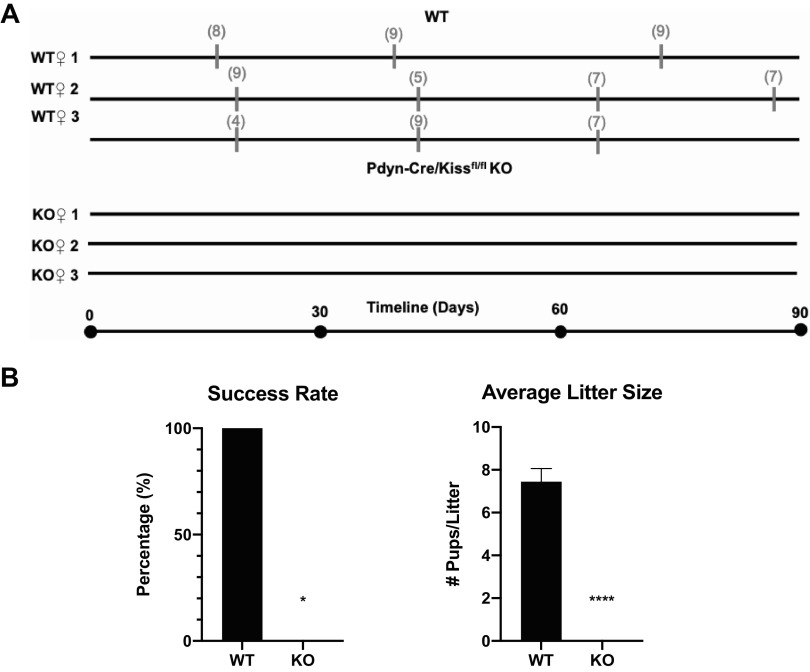
Fertility assessment of Pdyn-Cre/Kiss1^fl/fl^ knockout (KO) female mice. *A*: each virgin wild type (WT) or Pdyn-Cre/Kiss1^fl/fl^ KO female was paired with one proven, WT stud male each over a span of 90 days, beginning at female reproductive maturity. Gray thick marks indicate timepoints of litter births, with gray numbers in parentheses above indicating the number of live pups in that litter. Reference timeline is shown beneath all WT and KO individual fertility timelines (WT *n* = 3, Pdyn-Cre/Kiss1^fl/fl^ KO *n* = 3). *B*: Pdyn-Cre/Kiss1^fl/fl^ KO females demonstrate infertility having 0 litters in 90 days (unpaired Student’s *t* test, ****P < 0.001, KO vs. WT, WT *n* = 3, Pdyn-Cre/Kiss1^fl/fl^ KO *n* = 3), and a significantly reduced reproductive success rate at 0% (chi-square test, *P < 0.05 KO vs. WT).

After observing significant deficiencies in ovarian development, cyclicity, and LH pulsatility, we investigated the impact of these reproductive impairments on female fertility. We paired either a WT or Pdyn-Cre/Kiss1^fl/fl^ KO virgin female with a proven, stud WT male, and monitored for parity events within 90 days of continuous mating, only separating the females from stud males during pregnancy and after birth of a litter (Fig. 7*A*). One WT mouse gave birth three times in a 90-day period, producing a total of 25 pups with an average of 8.67 pups per litter. The second WT mouse gave birth four times, producing a total of 28 pups with an average of seven pups per litter. The third WT mouse gave birth three times, producing 20 total pups with an average of 6.67 pups per litter. Pdyn-Cre/Kiss1^fl/fl^ KO female mice (*n* = 3), however, did not produce any litters in the entire 3-mo period. Thus, the number of pups per litter and rate of successful pregnancies remained at 0 or 0% in Pdyn-Cre/Kiss1^fl/fl^ KO females (Fig. 7*B*).

### Pdyn-Cre/Kiss1^fl/fl^ KO Males Are Subfertile

**Figure 8.**
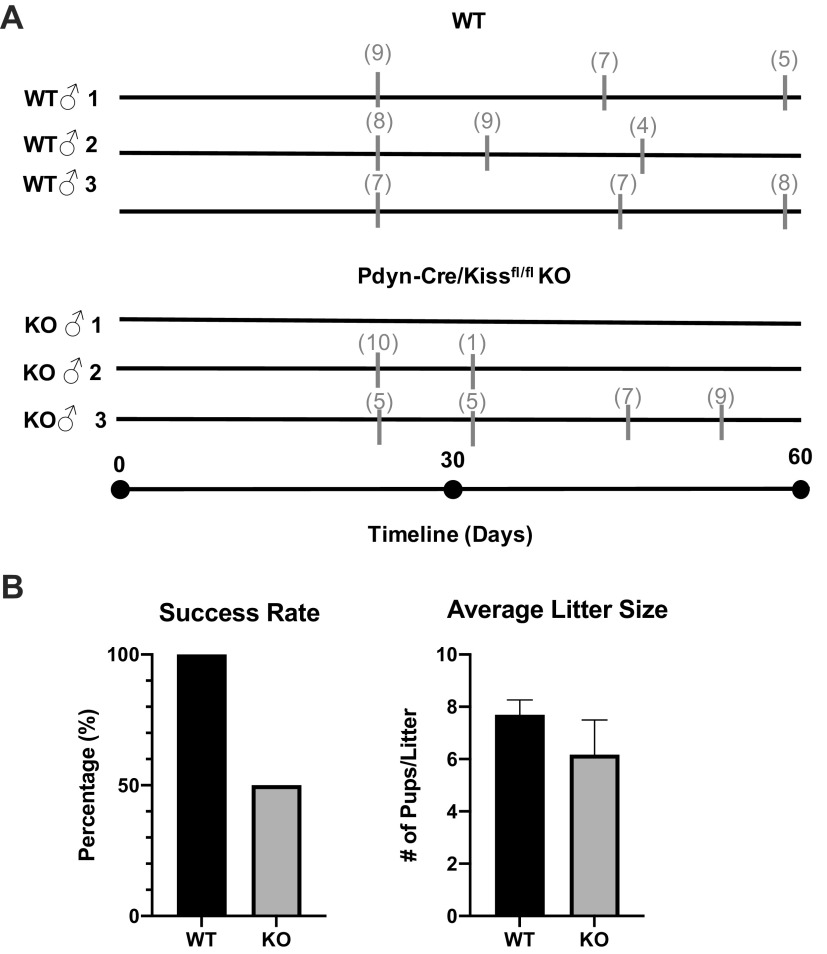
Fertility assessment of Pdyn-Cre/Kiss1^fl/fl^ knockout (KO) male mice. *A*: beginning at reproductive maturity, each virgin wild type (WT) or Pdyn-Cre/Kiss1^fl/fl^ KO male was paired with one proven, WT female for 10 days minimum before replacing the female with another proven, WT female. Individual, representative mice are shown in the timeline. Gray thick marks indicate timepoints of litter births, with gray numbers in parentheses above indicating the number of live pups in that litter. Reference timeline is shown beneath all WT and KO individual fertility timelines (WT *n* = 3, Pdyn-Cre/Kiss1^fl/fl^ KO *n* = 3). *B*: Pdyn-Cre/Kiss1^fl/fl^ KO males demonstrate variable, disrupted fertility with diminished, but no statistically significant difference in litter size (unpaired Student’s *t* test, WT *n* = 13, Pdyn-Cre/Kiss1^fl/fl^ KO *n* = 6) and reproductive success rate, which decreased to 50% (chi-square test, *P* = 0.1675 KO vs. WT, WT *n* = 5, Pdyn-Cre/Kiss1^fl/fl^ KO *n* = 3).

Upon observing a decrease in LH pulsatility and hypogonadism in Pdyn-Cre/Kiss1^fl/fl^ KO males, we sought to investigate male fertility status as well (Fig. 8). A virgin WT or Pdyn-Cre/Kiss1^fl/fl^ KO male mouse was paired with a proven fertile WT female mouse for a minimum of 10 days before replacing the female mouse with another proven WT female mouse. The litter size of successful pairings was recorded. There were no significant differences between the litter sizes of WT male-sired litters and Pdyn-Cre/Kiss1^fl/fl^ KO male-sired litters (unpaired Student’s *t* test, *P* = 0.2275), however the litter size decreased over time in some Pdyn-Cre/Kiss1^fl/fl^ KO animals. Fertility in the Pdyn-Cre/Kiss1^fl/fl^ KO male mice was highly variable, matching the variable spermatogenesis, and the reproductive success rate of Pdyn-Cre/Kiss1^fl/fl^ KO males compared with WT males was decreased by 50%, although the variability did not yield a significant difference (chi square test, *P* = 0.1675 KO vs. WT).

### Serum Estradiol Levels Were Undetectable in the Pdyn-Cre/Kiss1^fl/fl^ KO Female Mice

Pdyn-Cre/Kiss1^fl/fl^ KO female mice exhibited disrupted estrous cycles, thread-like uteri, infertility, and a striking hypogonadal phenotype, hence, we sought to evaluate their serum estradiol levels. Serum was collected from female mice during diestrus and pooled into two groups per genotype to be analyzed using LC-MS. The average estradiol level in WT female mice was 6.58901 ± 0.8967 pg/mL. In contrast, the estradiol level of Pdyn-Cre/Kiss1^fl/fl^ KO female mice was below the limit of detection of LC-MS (Table 2).

**Table 2.** Serum estradiol levels measured by LC-MS

Genotype	Diestrus E_2_, pg/mL
WT females	6.58901 ± 0.8967
Pdyn-Cre/Kiss1^fl/fl^ KO females	ND

Data are represented as average ± SE. Serum from wild-type (WT) (*n* = 7) Pdyn-Cre/Kiss1^fl/fl^ knockout (KO) (*n* = 7) was pooled into two groups per genotype to gain sufficient volume for LC-MS (*Group 1*: *n* = 3, *Group 2*: *n* = 4, for both genotypes). ND, not detected.

## DISCUSSION

In the present study, we have generated a novel mouse model targeting the *Kiss1* cell population in the ARC, to dissect the roles of ARC Kiss1, independent of its role in other CNS centers, in regulating the reproductive axis. This study provides the first direct evidence in a mouse model that KNDy neurons are the GnRH pulse generator. The accompanying studies show that deletion of *Kiss1* in the ARC results in a significant decrease in LH pulse frequency in both sexes, affecting gonadal development and gametogenesis, and resulting in infertility in Pdyn-Cre/Kiss1^fl/fl^ KO females and subfertility (ranging from infertility in one mouse to fewer litters in other mice) in Pdyn-Cre/Kiss1^fl/fl^ KO males. ARC *Kiss1* deletion was generated using a novel floxed *Kiss1* mouse line and the Pdyn-Cre mouse to embryonically delete *Kiss1* in the mouse KNDy neuron (Pdyn-Cre/Kiss1^fl/fl^). This targeting strategy was used since dynorphin has been shown to have conserved (∼94%) coexpression with ARC kisspeptin neurons (17, 18, 28) and although in situ hybridization (ISH) studies in the mouse showed that *Dyn* expression is present in 33% of AVPV kisspeptin neurons (less than 1/10th of the *Dyn* expression in ARC kisspeptin neurons) (18), immunocytochemistry (ICC) studies in the ewe showed no protein-level colocalization between dynorphin and kisspeptin in the AVPV, noting that ISH and ICC dynorphin expression levels differed significantly (17, 18, 47). ARC-specific knockout of kisspeptin was documented using qRT-PCR and immunofluorescence staining to assess *Kiss1* mRNA and protein expression in Pdyn-Cre/Kiss1^fl/fl^ KO mouse brain, respectively.

At the onset of puberty, a loss of estradiol negative feedback sensitivity on ARC kisspeptin secretion occurs to release the “brake” on kisspeptin stimulation on GnRH neuron activity. This mechanism invokes an increase in both ARC and AVPV kisspeptin, with an exaggerated AVPV response during the progression of puberty. The role of ARC kisspeptin in the pubertal process appears to be in the completion of pubertal progression to the point of reaching adult-pattern GnRH pulsatility and adult ovulatory cyclicity, rather than the initiation of puberty itself (48–50). This observation is consistent with the current study in which the onset of puberty was preserved in Pdyn-Cre/Kiss1^fl/fl^ KO male and female mice compared with control mice. Meanwhile, indicators of pubertal completion, such as adult-level GnRH/LH pulsatility and ovulatory cyclicity are compromised, consistent with observations in the literature (49, 51). In addition to supporting the literature, the preservation of puberty initiation further indicates the functional preservation of the estradiol positive feedback-sensing AVPV kisspeptin neurons, which appear critical for this phenomenon.

Furthermore, our data in young adult mice do not support a role for arcuate kisspeptin in body weight regulation, as other studies using a global knockout of *Gpr54* (now known as *Kiss1r*) or toxin-ablated or gene-targeted silencing of kisspeptin have posited (52). Previous studies have highlighted the synaptic connections of arcuate kisspeptin neurons with agouti-related peptide (AgRP)-, neuropeptide Y (NPY)-, and proopiomelanocortin (POMC)-expressing neurons, all implicated in energy homeostasis due to their shared lineage with ARC kisspeptin neurons (53–55). An ARC *Kiss1* loss-of-function mouse model, induced by AAV1-DIO-GFP:tetanus toxin (TeTx) viral stereotaxic injection into adult Kiss1-Cre female mice (Table 1), demonstrated an increase in body weight and obesity due to dysregulated feeding behavior (25). To examine whether this was a function specific to kisspeptin in KNDy neurons, we tracked body weight changes in WT and Pdyn-Cre/Kiss1^fl/fl^ KO mice from 21 to 49 days of age, finding no differences between genotypes in either sex. As toxin-based silencing in AAV1-DIO-GFP:TeTx mice was induced in adulthood, it is possible that disrupting ARC kisspeptin in fully matured mice may result in a sudden shift in the energy homeostatic circuit involving AgRP, NPY, and POMC, culminating in abnormal weight gain. Furthermore, toxin-based interventions may interrupt KNDy cell interactions with neurons involved in energy homeostasis. The ARC kisspeptin deficiency in Pdyn-Cre/Kiss1^fl/fl^ KO mice occurred embryonically, which may have allowed for developmental compensation of the energy homeostatic circuit in utero while maintaining cellular integrity.

Previous studies of toxin-ablated or gene-targeted silencing of ARC *Kiss1* in rodent models (Table 1), have shown that experimental female mice develop persistent diestrus or abnormal estrous cyclicity (25–27). Our findings align with the findings of these ARC-targeted kisspeptin deletion studies, providing strong evidence that ARC-specific kisspeptin is primarily responsible for the maintenance of regular estrous cyclicity (24, 56, 57).

Ideally, measuring GnRH secretion would involve frequent sampling directly from the hypophysial portal system. However, this method is not readily feasible in small mammals such as mice (41, 58). Since GnRH pulses and LH pulses have well-documented synchronization, we used LH pulsatile measurements as a proxy for GnRH pulsatile secretion (58, 59). Early studies in rats and monkeys with lesions in the ARC demonstrated the necessity of this region in maintaining normal LH pulsatility with the hypothesis that ARC *Kiss1* neurons function as the GnRH pulse generator, within which synchronization with neurokinin B mediates pulse initiation and dynorphin A mediates pulse termination (60, 61). Therefore, it was reasonable to predict that, with a loss of ARC *Kiss1*, there would be an elimination of GnRH/LH pulses. However, with specific deletion of *Kiss1* in our genetically modified mouse model, we observed a striking, significant decrease in LH pulse frequency in KO mice of both sexes, but not a complete elimination. LH pulse frequency remained at or ∼1 pulse per 3 h in Pdyn-Cre/Kiss1^fl/fl^ KO mice of both sexes. As evidenced by validation studies, it is unlikely that this is a consequence of incomplete kisspeptin protein elimination in the ARC.

Due to the embryonic onset of our KO, it is reasonable to assume developmental compensation may lead to recruitment of different neuronal populations or neuronal factors to support the partial retention of this phenomenon. For example, an upregulation in GABA or other neurotransmitters could contribute to increased GnRH firing and pulse generation, or result in developmental compensation that may be differentially regulated in this model (62, 63). Another possibility to consider is that neurokinin B, which is not directly targeted in the Pdyn-Cre/Kiss1^fl/fl^ KO mouse model, may compensate for GnRH/LH pulse induction to some degree by directly stimulating GnRH neurons (64). Similarly, extra-arcuate neuronal populations, such as the AVPV, may positively contribute to GnRH/LH pulsatility as suggested in previous anterograde and retrograde tracing studies (65). The percentage of ARC *Kiss1* neurons, relative to other neurons, that mediate the mechanisms underlying GnRH/LH pulsatility remains to be determined. A comparison to inducible ARC kisspeptin knockout in adult animals, however, may provide insight into this question as well as to the degree of developmental compensation that occurs at different timepoints.

Although not statistically significant, LH pulse amplitude was 42% lower in Pdyn-Cre/Kiss1^fl/fl^ KO females and 59% in Pdyn-Cre/Kiss1^fl/fl^ KO males. Interestingly, although mean LH was similar between WT and Pdyn-Cre/Kiss1^fl/fl^ KO groups of both sexes, AAV1-DIO-GFP:TeTx female mice exhibited a 40% decrease in LH (25). This difference, although not clearly understood, could be attributed to differences in the ARC kisspeptin targeting strategies or yet unidentified factors responsible for re-equilibrating average LH secretion. Overall, our findings confirm that timed regulation of GnRH in mice, and subsequent pulsatile LH release, is mediated by kisspeptin in KNDy neurons, rather than kisspeptin regulating the total amount of gonadotropin released.

Due to the persistent diestrus and impaired LH pulsatility observed in our Pdyn-Cre/Kiss1^fl/fl^ KO females, we explored the ability of these mice to ovulate. Primordial, primary, and secondary follicles formed before the antral stage, referred to as preantral follicles, develop independent of gonadotropins. After the antral follicle stage, a developmental switch allows folliculogenesis to progress in a gonadotropin-dependent manner (66, 67). We observed distinct and varied stages of folliculogenesis in WT ovaries including early-stage follicles, antral follicles, and corpora lutea in normal proportions representing the sequential stages of folliculogenesis. In contrast, Pdyn-Cre/Kiss1^fl/fl^ KO ovaries exhibited early-stage follicles and antral follicles, but no corpora lutea. The presence of antral follicles in Pdyn-Cre/Kiss1^fl/fl^ KO mice suggests that the temporal lag in gonadotropin secretion causes folliculogenesis progression to halt at antral follicle production. To thoroughly explore the lack of corpora lutea in KO ovaries, we counted total corpora lutea through sequential sectioning of whole ovaries and failed to find any corpora lutea in Pdyn-Cre/Kiss1^fl/fl^ KO mice. Counting total corpora lutea in whole ovaries is a method used to measure ovulation rate (40, 68), suggesting that not only does the deletion of ARC *Kiss1* result in arrested folliculogenesis at the antral follicle stage, but it may also inhibit ovulation. The arrested folliculogenesis phenotype was observed previously by our laboratory in GKirKO mice, which modeled the inhibition of kisspeptin signaling to GnRH neurons (37). Similarly, in a recent manuscript in which KNDy neurons were rescued in global Kiss1^−/−^ female rats by stereotaxic injection of *AAV-Kiss1* into the arcuate nucleus (Table 1), the authors noted a rescue in ovarian weight and mature ovarian follicles (24).

Normal cyclic alterations in uterine weight occur during the estrous cycle, such that during diestrus, the uterine horns are thinner, and during estrus/proestrus the uterine horns become engorged due to cellular proliferation required for implantation (69, 70). The decreased uterine weight observed in Pdyn-Cre/Kiss1^fl/fl^ KO mice is likely due to the predominant time spent in diestrus compared with their WT counterparts. This is confirmed by the undetectable levels of estradiol in the persistently diestrus Pdyn-Cre/Kiss1^fl/fl^ KO female mice when compared with the normal levels found in WT female mice during diestrus (71). All layers of the uterus are present in the Pdyn-Cre/Kiss1^fl/fl^ KO, however the total cross-sectional area of the Pdyn-Cre/Kiss1^fl/fl^ KO endometrial and myometrial layers were significantly decreased compared with WT uteri, attesting to the lack of cellular proliferation. These uteri exhibit a “threadlike” appearance—a term used to describe both the GPR54^−/−^ mice (2) and the GKirKO mice (37). Although the Pdyn-Cre/Kiss1^fl/fl^ KO uteri are noticeably thinner and “threadlike,” they do not exhibit differences in the overall length of the uterine horns, unlike the uteri observed by the GPR54^−/−^ and GKirKO mice. Therefore, the thin Pdyn-Cre/Kiss1^fl/fl^ KO uterus is likely due to a lack of the normal dynamic changes in estradiol secretion.

Although we observed arrested folliculogenesis in females, we observed variable impairment in spermatogenesis between WT and Pdyn-Cre/Kiss1^fl/fl^ KO males. One out of the four observed Pdyn-Cre/Kiss1^fl/fl^ KO males exhibited normal spermatogenesis, with the presence of mature spermatids in the lumen of the seminiferous tubules. The other three Pdyn-Cre/Kiss1^fl/fl^ KO males exhibited strikingly impaired sperm maturation, with few mature spermatids in the seminiferous tubules’ lumen. We confirmed the variable impaired spermatogenesis in Pdyn-Cre/Kiss1^fl/fl^ KO males when we conducted formal fertility testing on WT and Pdyn-Cre/Kiss1^fl/fl^ KO males when paired with proven, WT females. Of the Pdyn-Cre/Kiss1^fl/fl^ KO males tested, one exhibited complete infertility and no mature sperm were found. Another Pdyn-Cre/Kiss1^fl/fl^ KO male was able to sire only two litters before becoming infertile. In striking contrast to the first Pdyn-Cre/Kiss1^fl/fl^ KO male, but following the variable spermatogenesis phenotype, the last Pdyn-Cre/Kiss1^fl/fl^ KO male exhibited completely intact fertility and had 100% reproductive success. This phenotype is similar to an observation that was made in the GKirKO mice, in which male GKirKO mice exhibited defective spermatogenesis and had few mature sperm in the lumen of the seminiferous tubules (37). Thus, ARC-derived kisspeptin is critical for gametogenesis in male and female mice.

In addition to defects in gametogenesis, we observed a significant decrease in the size and weight of the ovaries and testes of Pdyn-Cre/Kiss1^fl/fl^ KO mice—a hallmark phenotype of hypogonadism. The smaller and lighter gonads in male and female Pdyn-Cre/Kiss1^fl/fl^ mice are similar to those of GKirKO mice which also exhibited hypogonadotropic hypogonadism (37). The decrease in serum FSH in Pdyn-Cre/Kiss1^fl/fl^ KO males and females is likely the causative factor for this hypogonadal phenotype. In both mouse models, ARC kisspeptin signaling to the GnRH neuron is lost, leading to strikingly similar phenotypes of hypogonadism and impaired gametogenesis.

Since the female Pdyn-Cre/Kiss1^fl/fl^ KO mice had arrested folliculogenesis, disrupted estrous cyclicity, decreased LH pulsatility, and hypogonadism, we hypothesized that fertility in these mice would be impaired. Indeed, none of the Pdyn-Cre/Kiss1^fl/fl^ KO females had given birth in continuous paired-housing with proven, stud WT males, whereas WT females produced pups at least three times during the 90-day study. This provides strong evidence that Pdyn-Cre/Kiss1^fl/fl^ KO female mice are infertile. Thus, the Pdyn-Cre/Kiss1^fl/fl^ KO female infertility is most likely due to the arrested folliculogenesis seen in these mice, stemming from impaired timing of LH pulses, hindering proper gonad development as well as the consistent production of mature ovulatory follicles.

There has been recent interest in investigating the therapeutic use of kisspeptin to restore physiological hormonal secretion in hypogonadal men and women. Kisspeptin has been used in studies of adults with functional or hypothalamic hypogonadism, leading to restoration of LH pulsatility, suggesting that the central basis for these disorders is abnormal kisspeptin secretion (72–75). However, no studies have been performed to confirm the singular, pathogenic role of kisspeptin or to localize this effect in the arcuate nucleus. Evidence from these studies suggests that hypothalamic amenorrhea, a form of hypogonadotropic hypogonadism, can be considered as a state of hypothalamic kisspeptin deficiency.

In summary, the Pdyn-Cre/Kiss1^fl/fl^ KO mouse line resulted in a conditional arcuate-nucleus specific deletion of kisspeptin and, for the first time, distinguishes the role of ARC kisspeptin neurons in the hypothalamic-pituitary-gonadal axis independent of AVPV kisspeptin. Our data, demonstrating the normal onset of puberty in the mice bearing a deletion of kisspeptin in the arcuate nucleus are strong evidences of AVPV kisspeptin’s important role in puberty. ARC kisspeptin neurons in female mice are shown to be primarily responsible for normal estrous cyclicity and, in both sexes, the primary regulator for GnRH/LH pulse generation. The striking disruption in temporal GnRH/LH pulsatility arrests folliculogenesis and results in female infertility. Although males with deletion of ARC kisspeptin have a similar, dramatic diminishment of GnRH/LH pulsatility, they have variable, impaired spermatogenesis that corresponds with their subfertility. This genetically modified mouse model provides novel evidence that ARC kisspeptin is responsible for GnRH/LH pulsatility, estrous cyclicity, and ultimately, the maintenance of normal fertility status in addition to gametogenesis in both sexes. This mammalian model of postpubertal central hypogonadism associated with disruption of GnRH/LH pulsatility provides evidence that arcuate kisspeptin is central to the abnormalities resulting in adult-onset hypothalamic hypogonadism.

## SUPPLEMENTAL DATA

Supplemental Table S1 and Supplemental Fig. S1: https://figshare.com/s/0019f2137f93b6f16a28.

## GRANTS

This study was supported by the Eunice Kennedy Shriver National Institute of Child Health and Human Development (NICHD)/NIH Grant R01 HD068777 (to S. Radovick, Multiple Principal Investigator) and University of Virginia Center for Research in Reproduction Ligand Assay and Analysis Core (NCTRI) Grant P50-HD28934.

## DISCLAIMERS

This article was prepared while A. Wolfe was employed at Johns Hopkins University. The opinions expressed in this article are the author’s own and do not reflect the view of the National Institutes of Health, the Department of Health and Human Services, or the United States government.

## DISCLOSURES

No conflicts of interest, financial or otherwise, are declared by the authors.

## AUTHOR CONTRIBUTIONS

N.N., A.L.N., A.W., J.E.L., and S.R. conceived and designed research; N.N. and A.L.N. performed experiments; N.N. and A.L.N. analyzed data; N.N., A.L.N., J.E.L., and S.R. interpreted results of experiments; N.N. prepared figures; N.N. drafted manuscript; N.N., A.L.N., A.W., J.E.L., and S.R. edited and revised manuscript; A.L.N., A.W., J.E.L., and S.R. approved final version of manuscript.
